# Lessons from the eradication of smallpox: an interview with D. A. Henderson

**DOI:** 10.1098/rstb.2013.0113

**Published:** 2013-08-05

**Authors:** D. A. Henderson, Petra Klepac

**Affiliations:** 1 Center for Health Security, University of Pittsburgh Medical Center, Baltimore, MD 21202, USA; 2 Johns Hopkins Bloomberg School of Public Health, Baltimore, MD, USA; 3 Department of Applied Mathematics and Theoretical Physics, University of Cambridge, Cambridge CB3 0WA, UK

**Keywords:** smallpox, disease eradication, interview, poliomyelitis, guinea worm

## Abstract

It has been more than 35 years since the last naturally occurring case of smallpox. Sufficient time has passed to allow an objective overview of what were the key factors in the success of the eradication effort and what lessons smallpox can offer to other campaigns. Professor D. A. Henderson headed the international effort to eradicate smallpox. Here, we present a summary of D. A. Henderson's perspectives on the eradication of smallpox. This text is based upon the Unither Baruch Blumberg Lecture, delivered by D. A. Henderson at the University of Oxford in November 2012 and upon conversations and correspondence with Professor Henderson.

## 1. Introduction

Before smallpox eradication was attempted, four other eradication efforts had failed [1–3]: namely; hookworm, yellow fever, yaws and malaria. The malaria eradication campaign was particularly extensive and intensive both in terms of manpower and financial resources (more than $2.5 billion expended from 1957 to 1975) [4]. These failures eventually led many in the global health community to shift their focus from targeted disease-eradication attempts to less explicitly defined programmes such as providing basic health services. There was a perception that eradication programmes, referred to as ‘vertical programmes’, could have a particularly deleterious impact in inhibiting the development of the basic health services (so-called ‘horizontal’ or ‘integrated’ programmes). Thus, a proposal to undertake a global smallpox eradication campaign was a divisive and politically charged issue, further complicated by the fact that the World Health Organization (WHO) was reluctant to support another eradication campaign. However, in 1966 the World Health Assembly (WHA) passed a resolution approving an annual budget of $2.4 million to support a 10-year smallpox eradication plan by the very narrow margin of two votes (58 were needed for approval, it received 60) [1].

## 2. Interview

### (a) How did the smallpox eradication programme start, and how was it received?

During the 1960s, expenditure for the malaria programme represented 20 per cent or more of all funds available to WHO, thus constraining other control programmes. By the late 1960s, it became apparent that the programme would be far more costly and take far longer than many anticipated. An increasingly prevalent view was that disease eradication was not possible (see Rene Dubos' book *Man Adapting* [5]). This was the view of the Director General of WHO in 1966, Marcelino Candau.

Principal direction of the malaria programme had been provided by an internationally recruited WHO staff of some 500–600 specialists. National malaria staff operated entirely separately from in-country basic health services. The heads of national malaria programmes reported to the heads of state, not to the Ministries of Health (MoH). Those who worked in the malaria programme were usually paid somewhat more than those in the basic health services, which enabled the recruitment of some of the best people into the malaria programme. Understandably, this had substantial negative repercussions on the development of health services in countries where the malaria programme was operating.

With the growing problems in the malaria eradication programme, it would seem unlikely that a proposal to undertake the eradication of a second disease would be well received. However, the proposal to eradicate smallpox originated from an unexpected source—the Soviet Union. The Soviet Union and several of its allies returned to participation in WHO (and other United Nations agencies) after years of absence. One of its first acts, in 1958, was to propose that WHO undertake a smallpox programme. Victor Zhdanov, a virologist and deputy minister of health for Russia, called on the WHA to undertake the global eradication of smallpox, even quoting the favourable views of Thomas Jefferson while doing so. At the subsequent Assembly, the proposal was approved by acclamation. Other countries had been pleased that the Soviet Union had decided to return to full participation in WHO and were anxious to exhibit a sense of solidarity. However, over the next 7 years, little progress was made. WHO allocated limited funds, and voluntary contributions by countries were sparse. In 1966, at the request of the Assembly, the Director General drew up and presented a 10-year plan. It had two components: (i) systematic vaccination and (ii) a new concept—surveillance and containment. The latter called for weekly reports of cases from all health facilities and containment of outbreaks by special containment teams. It called for a WHO budget contribution of $2.4 million per year; voluntary contributions were expected to supplement this. Many countries doubted that eradication was possible and others were reluctant to agree to increasing their budgetary assessment by the amounts that would be required. The eradication plan was put to a vote. The Assembly, which normally reaches decisions fairly quickly and by acclamation, debated for 3 days on this proposal, and in the end put it to a vote. The resolution was passed by the narrow margin of two votes.

### (b) How did the US get involved in the smallpox eradication efforts when it was initially opposed to it?

The US got involved indirectly. In the 1960s, I was at the US Centers for Disease Control (CDC) in charge of the surveillance section, mainly for viral diseases, smallpox, measles, flu, and so forth. I had approximately 40 staff; some were stationed at our Atlanta headquarters; others were in state and local health departments. In the early 1960s the National Institutes of Health (NIH) staff and Merck became interested in the possible use of a new Merck measles vaccine in African countries where measles was an often fatal disease. In the US, measles vaccine was given together with gamma-globulin to diminish the possibility of high fever sometimes associated with administration of measles vaccine. Use of gamma-globulin with the vaccine would not be feasible for countries with limited health services. Thus, they wanted to undertake a study of the effects of vaccine without immunoglobulin. An NIH-led study in Upper Volta (now Burkina Faso) showed that measles vaccine worked well without immunoglobulin and had few adverse reactions [6,7]. The government in Upper Volta supported the study in return for enough vaccine for all of its children, and US Agency for International Development (USAID) indicated its willingness to cover the costs. Upper Volta was a member of L'Organisation de Coordination et de Coopération pour la Lutte contre les Grandes Endémies (OCCGE), a consortium of nine former French colonies in western Africa that collaborated in providing preventive and health services. This organization pressed USAID and Merck to make measles vaccine available for all children in these countries during the course of a 4-year programme. It would require training national teams from each country. As the focus of NIH was research, and not field operations, the CDC was asked to assume this role.

I thought the programme was a bad idea. The programme was expected to vaccinate 25 per cent of the children each year after which the countries would be expected to bear the costs for continuing vaccination. At that time, the measles vaccine cost $1.75 a dose, but the countries could not even afford 10 cents per dose for yellow fever vaccine. Starting and then stopping a vaccination programme in this manner was bad public health practice. It occurred to us that if a smallpox vaccination programme were instituted, it could be sustained, as smallpox vaccine cost only 1 or 2 cents a dose. Thus, we decided to propose a combined smallpox eradication–measles control vaccination programme. This would at least leave some structure and sustainable activity for smallpox control as a legacy.

We believed we could stop smallpox transmission in particular countries but, with the many nomads moving through West Africa, a region-wide programme would be necessary for effective smallpox control and perhaps to stop transmission. Thus, we suggested to USAID that the proposed programme be extended to 18 countries. This would have to include Nigeria, which constituted 67 per cent of the population of the whole area. The budget we proposed, $35 million over a 5-year period, was substantially greater than the $7 million that USAID had expected to spend. Much to our surprise, the proposal was accepted in full by President Johnson as a special US contribution to a United Nations initiative called ‘International Cooperation Year’.

We were only six months into this programme when I was called to Geneva to help WHO draw up the Director General's plans for the global smallpox programme which was to be presented months later at the May 1966 World Health Assembly. With full support now of the 18 West African countries, a positive decision for WHO to proceed with the global plan became a certainty. Subsequently, the Director General decided that an American should direct the entire programme. Thus, in less than a year I had moved from director of a CDC surveillance programme to director of an 18-country smallpox eradication–measles control effort and finally to the position of Chief Medical Officer for the global smallpox eradication programme.

### (c) At what point did it become clear that you would make the 10-year goal to eradicate smallpox?

Initially, the 10-year plan was little more than a theoretical hope. We had very little to work with in the planning—the data on reported cases were very incomplete and we had almost no information on what countries were doing already as there had been few reports provided to the WHO. Hope gradually transitioned to expectation but confidence that eradication could be achieved was not there until 1975.

About 4–5 years into the programme, we had begun thinking that eradication within another 3–4 years might be feasible. The West African programme had proceeded so well and so rapidly that it stunned everyone. This was a group of countries that included those with the highest incidence of smallpox in the world but, at the same time, had the least developed communications and transportation systems and the least sophisticated health services. The plans that we had made were substantially more successful than we had anticipated. Most of East Africa also became smallpox free only a few years later. From 1967 to 1973, the number of smallpox endemic countries dropped dramatically—from 31 to only five—India, Pakistan, Bangladesh, Nepal and Ethiopia. South Asia was a formidable problem and a heavily populated area. Significant changes had to be made in the strategy; resources had to be augmented—both in cash and people. The concluding barriers—Ethiopia and Somalia—posed other problems. Ethiopia was engulfed in civil war; teams in Somalia were handicapped by government officials refusing to report cases until they became epidemics. But, finally, on 26 October 1977 the world's last case of smallpox was discovered in Merka, Somalia.

### (d) You suggested that surveillance-containment was not the ultimate magic bullet that alone made the programme so successful. Could you expand on this?

**Figure 1.**
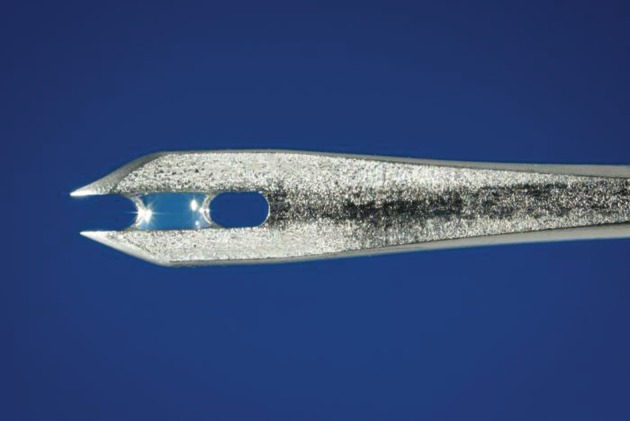
Bifurcated needle. Photo credit: CDC. (Online version in colour.)

A distinctive element of the programme, even more critical than surveillance-containment, was a heat-stable vaccine of assured potency, and a better technique for vaccination. Fortunately, Leslie Collier, of the Lister Institute, in the 1950s, had developed a commercially practical method for producing a freeze-dried vaccine that could withstand a temperature of 37°C for more than a month [8]. This was essential for all tropical areas. As we came to learn, however, it was in use in only a few of the more than 40 laboratories then making vaccine by some process. Quality control, if used at all, was to vaccinate some 8–10 rabbits or people and to use the vaccine if most developed some sort of lesion. With the help of a Dutch and a Canadian laboratory, we sought to obtain independent quality-control testing. The results were to show that for all vaccines intended for use in the programme in 1967 perhaps 80 per cent or more were seriously substandard and vaccine failures were frequent [9]. We recruited consultants from major laboratories to write a working production manual and they provided help and advice to laboratories in the developing countries. UNICEF provided commercial-sized freeze-drying machines. By 1973, all vaccine met potency, purity and stability requirements. Usage of a newly invented bifurcated vaccination needle (figure 1) in 1966 was another key element [10]. The needle was inexpensive, easy to use and required only a quarter of the vaccine dose normally required. Training of vaccinators required no more than 15 min and vaccinators could readily average 500 vaccinations per day obtaining more than 95 per cent successful takes.

A certain amount of vaccination was already being done by countries themselves, as smallpox was so greatly feared. Providing a fully potent, heat-stable vaccine made a huge difference and Kenya, Uganda, Tanzania and Malawi eliminated smallpox even before they could establish surveillance-containment programmes [6].

### (e) In India the surveillance-containment system had to be modified—how and why?

**Figure 2.**
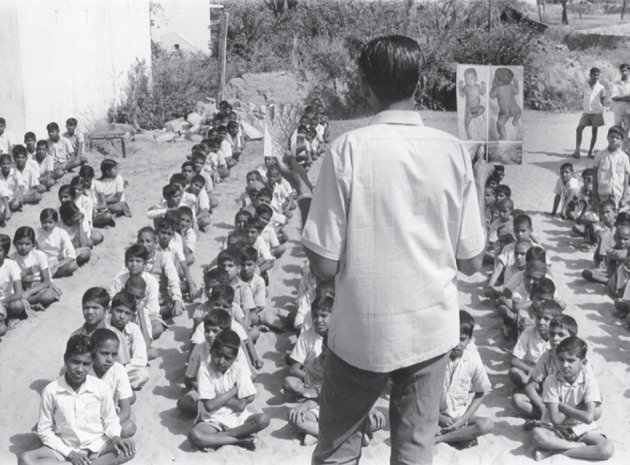
Schoolchildren and the Smallpox Recognition Card. Schoolchildren are being shown the WHO smallpox recognition card and asked if they know of cases. Information about cases within a 10 km (6.2 mile) radius was usually known to children who were between 8 and 12 years of age.

A basic component for smallpox surveillance was a weekly report from each health centre or hospital regarding each case of smallpox seen or a report stating that there were no cases. A team of two or three was then to go to the area, vaccinate all those in the immediate area and, in the process, search to see if there were other cases (figure 2 shows a team searching for cases). India provided for reporting from health units but the reports progressed through a hierarchy of offices, usually being delayed at each stop and sometimes changed. With a highly mobile population of 550–600 million, cases were usually reported too late for containment, if reported at all. More timely detection was essential. Accordingly, surveillance in India was augmented to focus on routine, repetitive active searches for cases—130 000 health personnel periodically swept through states searching every village and, eventually, every house during a 10-day period. The aim was to search 90 per cent of houses every two months to discover outbreaks and for containment vaccination teams to follow. Initially, the searches discovered tens or even hundreds of cases where none at all were being reported. With time, however, performance improved with the result that the last case in India was discovered little more than 18 months after the first search was undertaken [11].

### (f) You mentioned that in more sparsely populated areas, special search programmes were important for the surveillance-containment programme. What was the nature of these?

In low-density areas, one has the particular problem of having very few or no health personnel. However, as we discovered, there are always well-demarcated places where people regularly come together, such as markets or religious centres. In Ethiopia, people came to markets once a week, walking 10–15 miles to reach them. In Indonesia, we discovered the importance of schools—that it was possible for one worker to obtain a list of outbreaks over a wide area by going to schools and holding up a card showing a child with the typical rash of smallpox [12]. The children then told the questioner which villages, indeed which houses had smallpox. We came to learn that children between 8 and 12 years know almost everything that's going on in their own village and they are happy to share that information.

### (g) Is there any other disease that you would foresee being eradicated?

No. Not at this time given presently available relevant technologies, our understanding of the epidemiology of the infectious diseases, and pilot programmes of national disease elimination programmes. The one exception is guinea worm disease. A programme to eradicate it is progressing well, albeit it is in its 27th year, 13 years beyond the target date that had been set. Civil strife, however, continues to hamper it.

I believe that this is essentially the same answer that Frank Fenner, the world-renowned Australian virologist, and I provided in August 1980 at a meeting on eradication called at the Fogarty Center in Washington. The meeting was held only three months after the World Health Assembly meeting in Geneva had declared officially that smallpox had been eradicated. The mood of the 1980 meeting was a surprise to us both. We were well aware of the realities that we had encountered in smallpox eradication and the heroic efforts made by our own and national staff to achieve eradication. It was difficult to conceive of another disease problem that could similarly be addressed. However, the meeting theme was basically to decide *what next* should be slated for eradication. Frank Fenner and I were the keynote speakers, and both of us said, we did not think that at this point in time there was any candidate disease. Not surprisingly, the message was not welcomed. Subsequently, neither of us were invited to any of the many following meetings on eradication which have been conducted.

### (h) Can you tell us your views on the guinea worm programme?

Many of the lessons of smallpox eradication were directly translated into the guinea worm programme; in particular, a strong emphasis on reinforcing surveillance and feedback through quarterly reports, rapid response to outbreaks, coupled with regular rotations into the field by senior management personnel; as well as a considerable emphasis on community involvement. This includes educating the local people and the village chiefs about disease-prevention methods as well as the need for early identification and treatment. Filters for water purification and better water supplies also are making a difference. The programme is tied to the UNICEF provision of water programme, in order to accelerate the sinking of wells in many places. However, a major challenge for the eradication of guinea worm is civil disorder.

### (i) What do you see as being other benefits of the smallpox eradication programme?

First was the realization by health administrators in many countries that major improvements in the health status of its people could be effected even with small budgets and a paucity of well-trained health staff if goals were clear and steps were taken to involve local residents in the programme. Many weak, poorly managed primary healthcare programmes benefited from the smallpox programme, focusing, as it did, on greatly neglected vaccination initiatives. To achieve surveillance goals, weekly reports that provided feedback to field staff demonstrated a national interest in otherwise routine reports and improved morale of many in isolated primary care units. For example, the weekly smallpox surveillance programme in Brazil lead to the creation of the Brazil Weekly Epidemiological Report, a report that continues to be published today. Finally, it was the smallpox programme that provided the base and impetus for the launch of the follow-on Expanded Programme on Immunisation (EPI) whose genesis dates back to a first exploratory WHO meeting we had helped convene in 1970.

### (j) Can you tell us more about the Expanded Programme on Immunisation, its success in the Americas, and consequences in terms of belief in polio eradication?

The EPI was approved by the WHA in 1974. It emphasized the expansion of mobile vaccination efforts to include other vaccines in addition to smallpox—measles, polio and DTP (diphtheria, tetanus, pertussis). These vaccines had been thoroughly tested and were recommended for routine use in many countries. It would also represent an important initiative in emphasizing disease surveillance as a vital tool in understanding disease epidemiology, in monitoring progress in the programmes and in identifying problem areas and situations. The most successful of the programmes was in the Americas [13], where it was directed by Ciro de Quadros, the Pan-American Health Organization's (PAHO) programme director, a Brazilian and former senior WHO smallpox eradication advisor in Ethiopia. Success in controlling polio led countries of the Americas to agree to undertaking special EPI efforts that could lead to polio elimination. Surveillance networks were strengthened, a polio laboratory network was established, and a concept called ‘vaccination days’ was perfected [14]. This called for giving oral polio vaccine to all children under 5 years of age throughout an entire country on a single day. This began in Brazil and subsequently was replicated in many other countries [15,16]. A well-defined hemisphere-wide pattern of large-scale vaccination efforts, polio surveillance, field evaluations, provisions for laboratory diagnosis and inter-country cooperation, emerged in the Americas. In 1985, de Quadros proposed that polio eradication be established as a hemisphere-wide goal and this was approved by the PAHO Directing Council. The goal was to have the last case by 1990. The goal was missed—by about eight months—but a blueprint had been developed and subsequently thoroughly field-tested.

Progress in the elimination of polio in Latin America provided an incentive for a global programme called for in the Declaration of Talloires in 1988 [17,18] and approved later that year by the WHA. It was an emotional rather than a rational decision. There was no plan or budget. The principal proponents included the US delegation (technical advisors being primarily CDC staff), de Quadros, Albert Sabin, the creator of the oral polio vaccine, Rotary International that had committed financial support for vaccine purchase, and James Grant, the Director of UNICEF.

Concern about polio was entirely that of the industrialized countries with the US playing a predominant role. During the 1940s and 1950s, many infectious diseases faded in importance in the industrialized world. Antibiotics became available, housing, sanitary and nutritional standards improved and DTP vaccine came into use. Many infectious diseases declined to the point of being minor problems. Meanwhile, the incidence of polio increased, as did survivors who were left with visible and handicapping paralyses. Concern about polio increased sharply. With the support of President Franklin Roosevelt, himself a partly paralysed survivor, a special foundation called the National Foundation for Infantile Paralysis was created to raise funds for treatment and research. It was remarkably successful and it was responsible for funding the research laboratories that created polio vaccine. A vaccine comprised of inactivated polioviruses was perfected by a virologist, Jonas Salk, and began to be widely used beginning in 1955 [19]. It was given by injection. A second vaccine, comprised of living polioviruses that could be given by mouth, was developed by Albert Sabin. It became available in 1962 [20]. The oral vaccine soon became the more widely used because it was so easy to administer and inexpensive. It was especially suited for countries where health services were less developed. It was readily accepted in all countries [21].

In virtually all countries, oral vaccine rapidly replaced the inactivated vaccine, which could only be given with syringe and needle. Large-scale vaccination programmes using the oral vaccine were widely promoted in Latin America and a number of other developing countries. The number of cases of polio decreased rapidly. From the time of first use of the oral vaccine, Sabin actively promoted its widespread use in campaigns. As was discovered, such campaigns could be reasonably easily organized and were popular with politicians and the public. Rotary International took up the challenge of making the vaccine more readily available and pledged to raise $100 million by 2005—the 100th anniversary of its founding.

Sabin was especially articulate and insistent on the concept of intensive mass use of his vaccine throughout a country and demonstrated the disappearance of polio in Cuba following just such a programme [22]. Other programmes in the Americas led to the decision in 1985 to eliminate polio from the Americas by 1990. It was on this wave of enthusiasm that a global programme was decided in 1988.

In the discussions about global polio eradication, there were those who questioned the investment pointing out, correctly, that polio was not a major problem in the developing countries—that it certainly was not ranked among the top 20 or 25 disease problems in most. Accordingly, it was proposed that the bulk of the additional cost (above expenditures for the EPI) be borne by the industrialised countries, as they were the ones who would most greatly benefit. Potential donors pointed out that the provision of special polio funds for vehicles, personnel, etc. would flow through the EPI thus indirectly strengthening that programme.

### (k) Can you detail some polio-specific problems, and post-elimination issues?

The oral polio vaccine (OPV) has several characteristics that were not recognized until many years after licensure. However, from the time of licensing of OPV more than 50 years ago it was clear that the vaccine strains are somewhat unstable genetically and could occasionally revert to virulent form. One case of paralytic disease in a vaccinee or close contact occurred in every one to three million vaccinees [23]. This did not deter the use of the vaccine because the benefits of immunity from vaccination considerably exceeded the risk of acquiring the disease. As the number of cases diminished, this complication became of increasing concern.

Wholly unrecognized at the time of licensure was the fact that the poliovirus could combine with certain strains of another virus (Coxsackie) and exhibit other characteristics of persistent growth and spread [24]. The vaccines are referred to as recombinant strains or vaccine-derived polioviruses (VDPVs). These have been shown to be excreted long-term by immunocompromised persons [25] and even healthy individuals [26], and can lead to outbreaks of polio [23]. While vaccine-derived type 1 and type 3 polioviruses only rarely cause small outbreaks and seem to die out in the environment [27,28], type 2 poliovirus is puzzling. A case of the wild type 2 strain has not been seen since 1998. However, the recombinant type 2 vaccine strain has caused unexpected problems in causing cases and continuing to spread. The largest outbreak of recombinant type 2 poliovaccine virus in Nigeria included 278 confirmed cases from January 2005 to June 2009 [29]. Some have suggested that the attack rate and the severity of the disease caused by the recombinant strains are similar, if not identical, to those caused by the wild polio viruses [29] but this needs to be confirmed by more complete epidemiological data than has yet been made available (see [30]).

Furthermore, wild polioviruses can circulate undetected, despite surveillance, for up to 5 years, as has happened at least once (in Sudan [31]). Thus, certification of eradication will require at least 5 years with continued control and surveillance but using OPV during this time increases the risk of circulating recombinant vaccine viruses. How important this may be is uncertain as the recombinant strains of types 1 and 3 seem to die out spontaneously and after only a brief spread. How long a type 2 recombinant may survive is unknown. Only careful epidemiological–virological studies will tell. Consideration has been given to substituting inactivated polio vaccine for the oral vaccine but this is at least 20 times more expensive per dose and must be administered percutaneously and so requires more manpower and needles/syringe. It is impractical for use in slum areas where immunity after the OPV results from local spread of the live vaccine virus to the unvaccinated.

It is difficult at this time to visualize a feasible, affordable and supportable ‘endgame’ to achieve polio eradication as it is now defined—the complete cessation of transmission of all wild poliovirus strains and vaccine-derived strains.

### (l) Why were you sceptical about polio/other eradication efforts?

A simple answer is to point out that I had spent 11 years of my life endeavouring to eradicate smallpox and I knew well the diverse array of problems that a programme has to navigate. Smallpox eradication proved to be infinitely more difficult than I or anyone else had imagined it would be. Indeed, it is all but a certainty that any of a number of obstacles could have blocked its completion at various points in the programme but fortunately, in each case, unexpected events or special measures intervened to resolve problems.

Little had I appreciated the magnitude or number of other imponderables—floods, wars and famines, hundreds of thousands of refugees, national bureaucracies and constraints that rivalled the US in number and complexity, a difficult USAID programme (an unwilling but a significant contributor), and a sclerotic WHO administration that often thwarted or actively impeded what appeared to be logical initiatives.

There were other unexpected events—changes in governments, fortuitous laboratory discoveries, unexpected successes in launching vaccine production operations in developing country laboratories, and the emergence of needed leadership and courage by national and international staff at numerous critical points. The programme was ultimately successful but success hung in the balance on many occasions.

From my examination of the characteristics and needs for eradicating other diseases, each has prominent features that makes it far more difficult than smallpox. With smallpox, we had a vaccine so heat stable that teams travelled in the field without refrigeration devices. We had a vaccine that provided long-term protection with one dose. One could ascertain whether vaccination was successful by determining whether or not a pustule had developed at the vaccination site. There were no patients with subclinical infections. Thus, we could readily identify infected areas and contain the outbreaks. Cases were so typical and so readily identified that special diagnostic laboratories were unnecessary.

There are very few eradication enthusiasts who have had real-world practical experience in executing a successful component of an eradication programme at the local or national level and there are even fewer who have had the opportunity (or taxing challenge) of dealing with the practical and political complexities of a targeted programme at national and international levels. Prospects for eradication appear far more optimistic from the vantage point of a laboratory or an office in a university ivory tower.

### (m) The original target established by the WHA was to eradicate polio by 2000. Since then, the Global Polio Eradication Initiative had a number of extensions to this deadline. What is different about the latest extension?

Four previous 3 year extensions for the target date of polio eradication have previously been announced. These occurred in 2000, 2004, 2007 and 2010. Each called for an ever more intensified, more heavily supported universal effort but none succeeded. A fifth further intensified effort was announced in 2013. National and international leaders have pledged and are providing their full support, Bill Gates and the Gates Foundation have made polio eradication a special cause. It is recognized that the success of polio eradication has important implications for the successful and expanding global EPI.

### (n) Why do you think eradication programmes are so popular?

There is a belief that unless eradication is set as a goal, governments will not be willing to contribute the necessary resources for infectious disease control. To me, this seems like a weak excuse for inadequate efforts on the part of public health staff to educate and to persuade.

## 3. Final words

The guinea worm programme, as well as smallpox eradication, offer important prototypical examples. They have steadily evolved over time in response to the practical realities encountered in the field, the development of new approaches through research, the active involvement of local leadership and peoples, and imaginative quality control measures and supervision. Each developed different approaches and strategies adapted to local conditions. The polio programme had similar successes in eliminating polio from the Americas during the period 1985–1991 [32]. But the challenges in geography, populations and infrastructure of Asia and Africa have been overwhelming. The programme has struggled to develop satisfactory surveillance programmes for an elusive disease which has countless sub-clinical infections and which requires a sophisticated virus laboratory for confirmation of cases. An equally formidable obstacle has been the challenge of providing hundreds of millions of doses of a fragile, heat-labile vaccine throughout vast tropical and sub-tropical areas with limited health and transportation infrastructures. That not one but multiple doses of vaccine are required to provide assured immunity makes the task even more forbidding. Nevertheless, the number of infected areas has gradually been constricted, well-trained and motivated staff are working in the field, and resources far beyond any ever committed to control of a disease are being steadily made available. Hope is expressed that transmission of wild poliovirus will be stopped by the end of 2015, that circulation of recombinant poliovaccine viruses will be able to be stopped as well within the following 2–3 years. Success would be welcomed indeed by everyone.

Whatever the outcome, experiences to date with eradication programmes should be cautionary to any who contemplate an eradication effort. Disease control and elimination programmes can be varied in intensity and duration. Their success or failure is usually of limited importance to other countries. Eradication is a different story. It represents a global commitment from which it is problematic for countries to withdraw however unimportant the disease may be nationally. As investments in a programme grow, the penalty for failure is perceived to be ever greater. How much should be expended and at what cost financially and to other programmes? The polio programme is now in the 25th year of what was intended to be a 12-year effort.

From experiences to date with eradication programmes, it seems apparent that at least four factors should be in place before a launch: a reasonably thorough plan, an established research programme, success in a significantly large demonstration site, and a firm commitment by a majority of countries with definitive concerns and resources to support a programme. In light of the fact that there has been only one success among the seven global eradication programmes launched to date, the implications of possible failure should be clearly stated as well.
